# Variations of Thiol–Disulfide Homeostasis Parameters after Treatment with H1-Antihistamines in Patients with Chronic Spontaneous Urticaria

**DOI:** 10.3390/jcm10132980

**Published:** 2021-07-02

**Authors:** Clara Matei, Simona Roxana Georgescu, Ilinca Nicolae, Corina Daniela Ene, Cristina Iulia Mitran, Madalina Irina Mitran, Mircea Tampa

**Affiliations:** 1 Department of Dermatology, Carol Davila University of Medicine and Pharmacy, 020021 Bucharest, Romania; matei_clara@yahoo.com (C.M.); tampa_mircea@yahoo.com (M.T.); 2 Department of Dermatology, Victor Babes Clinical Hospital for Infectious Diseases, 030303 Bucharest, Romania; 3 Department of Nephrology, Carol Davila University of Medicine and Pharmacy, 020021 Bucharest, Romania; koranik85@yahoo.com; 4 Department of Nephrology, Carol Davila Clinical Hospital of Nephrology, 010731 Bucharest, Romania; 5 Department of Microbiology, Carol Davila University of Medicine and Pharmacy, 020021 Bucharest, Romania; cristina.iulia.mitran@gmail.com (C.I.M.); madalina.irina.mitran@gmail.com (M.I.M.); 6 Cantacuzino National Medico-Military Institute for Research and Development, 011233 Bucharest, Romania

**Keywords:** thiol, disulfide, urticaria, H1-antihistamines

## Abstract

Background. The pathogenesis of chronic spontaneous urticaria involves metabolic, immunological, and psychological factors. The thiol–disulfide exchange reactions could be a mechanism to counteract oxidative stress in patients with chronic spontaneous urticaria. Objective: The assessment of thiol–disulfide homeostasis parameters (TDHPs) according to disease severity and the influence of H1-antihistamine therapy in patients with chronic spontaneous urticaria. Material and method. We have included 30 patients with chronic spontaneous urticaria in the study and we have determined the levels of native thiol, total thiol, disulfides as well as the disulfide/native thiol ratio, disulfide/total thiol ratio and the native thiol/total thiol ratio, before and after therapy with H1-antihistamines. Results. The results of the study showed altered levels of TDHPs and their normalization after treatment with H1-antihistamines in patients with chronic spontaneous urticaria. We determined a statistically significant increase in the serum levels of total thiol, native thiol, and native thiol/total thiol ratio and a significant reduction in the levels of disulfides, disulfide/native thiol ratio and disulfide/total thiol ratio after treatment with H1-antihistamines. The normalization of the serum levels of TDHPs has been associated with the relief of symptoms and reduction or resolution of pruritus and urticarial plaques. Conclusion. These results suggest the involvement of thiol–disulfide homeostasis in the defense against the harmful effects of reactive oxygen species in patients with chronic spontaneous urticaria and the potential role of TDHPs in monitoring H1-antihistamine therapy. To the best of our knowledge, this is the first study investigating TDHPs in patients with chronic spontaneous urticaria before and after treatment.

## 1. Introduction

Various biochemical markers are currently employed to define oxidative stress in a biological system. Recently, a new molecular marker that provides quantitative results about redox processes has been investigated and approved to be used. It is about the analysis of thiol/disulfide homeostasis parameters (TDHPs) in biological samples, performed through the spectrophotometric method [1,2]. Plasma thiols can be divided into two groups, protein and non-protein thiols. SH-groups in the structure of proteins (protein thiols) are essential for their stability and functionality. Non-protein thiols include low molecular weight molecules, such as cysteine, homocysteine, glutathione (gamma-glutamyl-cysteinyl-glycine), gamma-glutamyl-cysteine, and cysteinyl-glycine [3,4,5,6]. Plasma thiols are physiological agents that neutralize free radicals and other reactive oxygen species (ROS). Thiols modulate the activity of the glutathione-associated enzymes and represent 52.9% of the total serum antioxidant capacity [7]. Thiols are the first antioxidants consumed under oxidative stress conditions. They react with ROS to form disulfides [8,9]. The exchange rate between the two molecules, thiol and disulfide, is modulated by the expression and activity of thiol–disulfide oxidoreductases (TDORs), enzymes that are involved in the formation, rearrangement, and cleavage of disulfide bonds [10,11]. Thiol–disulfide homeostasis (TDH) is important in various physiological processes, such as epidermal differentiation, cell apoptosis, antioxidant protection, etc. [1,3,8,12,13,14].

There are many factors that disturb TDH in extracellular spaces. The main processes that affect TDH are: oxidative stress, the rate of thiol–disulfide exchange, expression and activity of TDORs, export of thiol-containing molecules (glutathione, albumin) from the liver to extracellular spaces, the oxidation rate of thiols by ROS and the repair processes, availability and short half-life of thiols, regulation of amino acid transport systems between intracellular and extracellular compartments, lifestyle, hormonal status, smoking, etc. [10,15,16,17,18,19].

Recently, the alteration of TDH has been investigated in some skin disorders, such as telogen effluvium [20], vitiligo [12,21], rosacea [8], psoriasis [19,22], alopecia areata [23], basal cell carcinoma [24], fungal skin infections [25], atopic dermatitis [26,27], lichen planus [7,28], acne vulgaris [29], and urticaria [30,31,32].

The main characteristic of urticarial lesions have is an etiopathogenic substrate with plasma extravasion due to an increased vascular permeability (edema), vasodilation, and release of a large number of mast cells and basophil mediators (erythema), and stimulation of free nerve endings by chemical mediators (pruritus). The disease evolves with periods of exacerbation and spontaneous remission, often with unknown trigger factors. The etiopathogenic mechanisms in urticaria remain incompletely elucidated and include the interference between immunity, inflammation, oxidative stress, and neuroendocrine status [33,34,35]. Currently, in the medical literature, there are a few studies investigating TDHPs in patients with urticaria [30,31,32]. However, none of them has investigated TDHPs before and after treatment.

In this paper, we aim to investigate: (1) TDHPs in patients with chronic spontaneous urticaria according to the severity of the disease; (2) the influence of H1-antihistamines on TDHPs in patients with chronic spontaneous urticaria.

## 2. Materials and Methods

### 2.1. Study Participants

Of 84 patients who addressed the dermatology clinic of “Dr. Victor Babes” Hospital, with different forms of urticaria, we enrolled 30 patients with chronic spontaneous urticaria. We included patients with adequate nutritional status, without therapy with antihistamines, corticosteroids, immunosuppressants, nutritional supplements, with negative results of cutaneous intradermal autologous serum skin test (ASST), prick test, and skin provocation test.

After anamnesis and clinical examination, the urticaria activity score (UAS) was calculated by summing the pruritus intensity score (0—absent, 1—mild, 2—moderate, 3—intense) and the number of urticarial plaques (0—absent, 1—mild eruption with less than 20 papules or plaques/24 h, 2—moderate eruption with 20–50 papules or plaques/24 h, 3—severe eruption with more than 50 papules or plaques/24 h or large placards/24 h). The maxim value of UAS score is 6. All study participants gave their consent to the use of their biological samples in research studies; the study protocol was approved by the Ethics Committee of “Dr. Victor Babes” Hospital (2/15.02.2018). All the procedures and the experiments performed in the study respect the ethical standards in the Helsinki Declaration.

The patients were clinically and paraclinically evaluated in the pre-therapeutic phase (30 patients) and 6 weeks after the initiation of treatment (26 patients). The patients were treated with antihistamines in monotherapy or combination, at variable doses, depending on the severity of the disease.

### 2.2. Laboratory Tests

Blood samples were collected in the morning and then centrifuged at 1500× *g* for 10 min. The serum samples were stored at −80 °C. TDHPs were determined using the spectrophotometric method (HumaSTAR 300, GmbH, Wiesbaden, Germany). We used sodium borohydride (NaBH4, 10 mM) in order to transform the reducible disulfide into free functional thiol groups bonds, according to the reaction: R-S2-R‘ + NaBH4 → 2 R-SH + BH3 + Na. We used formaldehyde (10 mM, pH 8.2) to remove the amount of NaBH4, which was not used in the reaction. The levels of native thiol (NT) and total thiol (TT) were measured using 5,5′-dithiobis-2-nitrobenzoic acid (DTNB, 10 mM) according to the reaction: R-SH + DTNB → R-TNB + TNB. Half of the difference between TT and NT was considered the disulfide (DS) level. The results were expressed as μmol/L serum. The disulfide/native thiol ratio (DS/NT), disulfide/total thiol ratio (DS/TT), and native thiol/total thiol ratio (NT/TT) were calculated as follows:

- DS/NT (-S-S- * 100/-SH);
- DS/TT (-S-S- * 100/-SH + -S-S-);
- NT/TT (-SH * 100/-SH + -S-S-);
- NT: (-SH); TT: (-SH + -S-S-); DS: (-S-S).

### 2.3. Statistical Analysis

We analyzed the data for normality using Kolmogorov–Smirnov test. The comparison of experimental data between the two groups was carried out using the non-parametrical Wilcoxon signed-rank test. The relationship between pairs of two parameters was assessed by Spearman’s correlation coefficient (rho). We chose a significance level (*p*) of 0.05 (5%) and a confidence interval of 95% for hypothesis testing.

## 3. Results

Of the 84 patients who addressed our clinic with different forms of urticaria, 30 adult patients (mean age 29.73 ± 3.19 years) diagnosed with chronic spontaneous urticaria were selected. Of these, 18 (60%) were women, and 12 (40%) were men. In addition, seven of them (23.3%) had a positive history of angioedema (Table 1).

**Figure 1:**
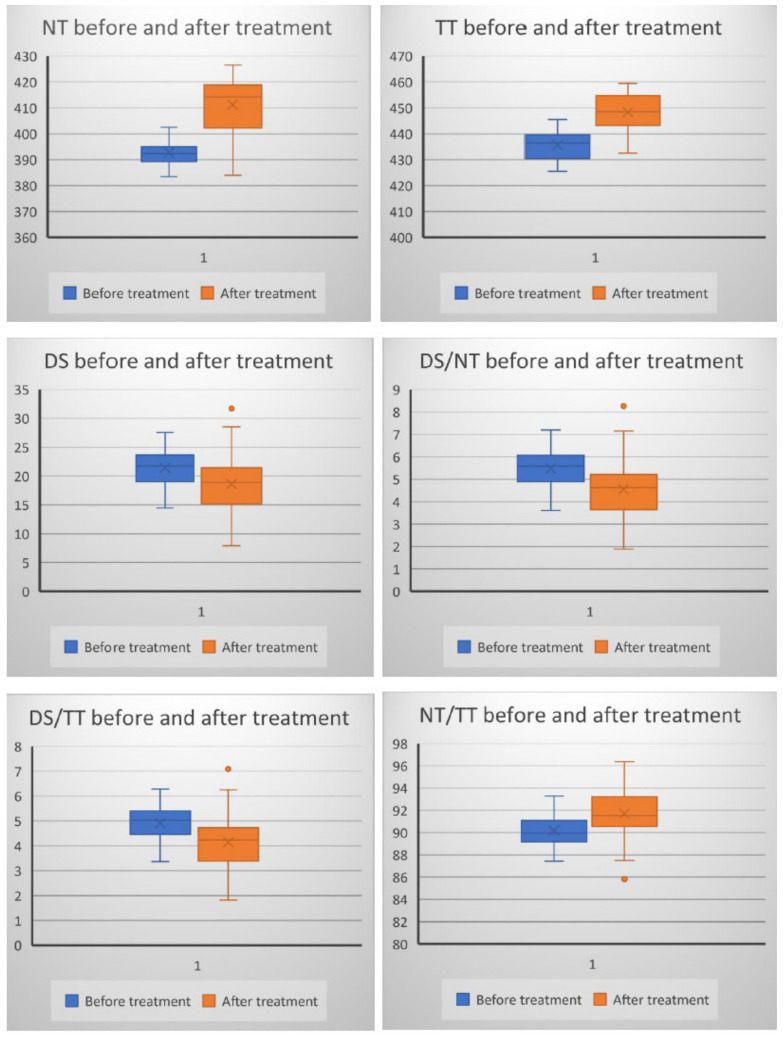
The evolution of TDHPs after treatment in patients with chronic spontaneous urticaria (X on the plot area represents the mean value).

The extent of urticaria was investigated by evaluating UAS. The mean value of UAS was 5.2 ± 0.76 before treatment and 0.69 ± 0.73 after treatment with H1-antihistamines (*p* < 0.05). The levels of NT and TT were higher (*p* < 0.01), and the levels of DS were lower (*p* < 0.01) after treatment with H1-antihistamines. There were also significant differences when compared DS/NT, DS/TT, and NT/TT ratios before and after treatment (*p* < 0.01) (Figure 1).

There was a statistically significant increase in the levels of NT (4.9%), TT (3.1%), NT/TT ratio (10%), and a significant reduction in the levels of DS (8.7%), DS/NT ratio (8.3%), DS/TT ratio (8.4%) after treatment (Figure 1).

In patients with chronic spontaneous urticaria, the relationship between TDHPs and the severity of the disease was analyzed both before and after treatment. There was a statistically significant negative correlation between UAS and NT, TT, NT/TT ratios before treatment as well as after treatment. A statistically significant positive association was recorded between UAS and DS/NT ratio, DS/TT ratio, before and after treatment (Table 2).

## 4. Discussion

It has been shown that TDH plays a critical role in many processes [1,2,3,8,12,14,36]. Under oxidative stress conditions, -SH groups convert to disulfides. Disulfide formation is the earliest sign of protein oxidation [15,37]. In turn, functional disulfide bonds can be reduced to thiol groups. The enzymatic-mediated interconversion between the reducible and oxidable thiols maintains TDH in a biological system. DS/NT ratio is the best marker for TDH. The levels of NT and TT and NT/TT ratios define the serum antioxidant potential, and the levels of DS and DS/NT and DS/TT ratios define the prooxidant status. The reduced levels of NT (-SH) and TT (-SH + -SS) show that these compounds are consumed as a consequence of oxidative stress generated in a particular pathological context [3,38,39]. Increased levels of thiols and decreased levels of DS are associated with proliferative diseases, while low levels of thiols and high levels of DS are associated with degenerative diseases [2]. In our study, we have shown that there is a relationship between thiol metabolism and the severity of urticaria, and the treatment with H1-antihistamines improves TDH in patients with chronic spontaneous urticaria. Decreased serum levels of NT and TT in patients with chronic spontaneous urticaria in the active (pre-therapeutic) phase may be explained by several mechanisms, such as reduced cysteine availability and glutathione (GSH) depletion [31], prolonged ROS generation [40], rapid formation of biologically active disulfide bonds, and irreversible conjugation of GSH [30]. The progressive increase in thiol levels in patients with chronic spontaneous urticaria in the clinical remission phase (post-therapy) denotes a reduction in ROS formation and an increase in the catalytic cleavage of active redox disulfide bonds. As a result, the alterations in thiol levels revealed in patients with chronic spontaneous urticaria may be attributed to the oxidative mechanisms. Mast cells are key players in the pathogenesis of urticaria. Mast cells degranulation initiates the inflammatory process. In addition, in chronic urticaria, an intradermal infiltration consisting of mast cells, CD4 T cells, neutrophils, eosinophils, and basophils has been observed. These cells are chronically activated and produce high levels of ROS [40]. These results reinforce the observations from previous studies on the contribution of oxidative stress to the pathogenesis of chronic urticaria [40,41].

In the medical literature, the results regarding TDHPs in patients with urticaria are contradictory. In a study performed on patients with acute urticaria (53 cases) and chronic urticaria (57 cases), the authors identified higher levels of NT, TT, and DS in patients with chronic urticaria and similar levels in those with acute urticaria compared to controls [30]. The study by Akdag et al. has revealed lower serum levels of NT and TT and higher serum levels of DS in children with chronic urticaria [31]. In another study on patients with acute urticaria, the serum levels of NT and TT, as well as DS, were lower in patients compared to the control group [32]. Abnormalities of TDH in dermatologic disorders could be associated with ischemia-modified albumin generation, high susceptibility to oxidative stress, altered adaptative response to chronic inflammation [30,31], impairment of antioxidant defense, changes in barrier functions, modified keratinocyte turnover rate, abnormal cellular redox regulatory network in cells and tissues, and alteration of processes, such as detoxification, cell growth, cell signaling, and apoptosis [42,43,44].

In recent years numerous studies which focused on TDH in cutaneous diseases have been performed. Demirseren et al. found a statistically significant difference between patients with basal cell carcinoma (34 cases) and controls (30 cases) regarding the levels of NT and DS, as well as DS/NT ratio and NT/TT ratio. The authors concluded that TDH may be a factor involved in the pathogenesis of basal cell carcinoma [24]. The studies that analyzed TDHPs in patients with psoriasis have revealed contradictory results. The serum levels of NT, TT, and DS were evaluated in 29 patients diagnosed with psoriasis vulgaris and 30 healthy controls. Plasma concentrations of DS were higher in patients with psoriasis vulgaris compared to the control group, and the levels of NT and TT, as well as the DS/NT ratio and DS/TT ratio, were similar in both groups. PASI score was not significantly correlated with any analyzed parameter. Elevated levels of DS indicate a thiol/disulfide imbalance in patients with psoriasis vulgaris due to oxidative stress and inflammation. In addition, the levels of DS did not show a significantly positive correlation with the severity of psoriasis [19]. Other authors who monitored 80 patients with psoriasis and 80 healthy subjects found similar serum levels of DS and lower serum levels of NT and TT in psoriasis patients compared to controls [45].

The evaluation of TDHPs in 50 patients with rosacea versus 42 controls revealed significantly higher levels of DS in rosacea patients. DS/NT ratio and DS/TT ratio were significantly higher, while NT/TT ratio was significantly lower in rosacea patients compared to controls. In conclusion, DS could be considered an indicator of oxidative stress in rosacea [8]. TDHPs have also been evaluated in patients with acne vulgaris. TT levels and DS/TT ratio were significantly lower in acne vulgaris patients (74 cases) than in controls (60 cases). The levels of NT, TT, and DS and the DS/NT ratio, DS/TT ratio, and NT/TT ratio did not differ significantly according to the clinical severity of the disease [29]. In a study on patients with telogen effluvium (52 cases) there were no statistical differences in the levels of DS, NT, and TT compared to controls (46 cases). It was concluded that TDH was not altered in patients with telogen effluvium [20]. A recent study on 100 patients with alopecia areata revealed that the levels of TT and NT and the NT/TT ratio were lower compared to controls, while the levels of DS and the DS/NT ratio and DS/TT ratio were significantly higher compared to the control group. These findings may explain the diffuse destruction of the hair follicle in the pathogenesis of alopecia areata [23].

The levels of NT and TT were found to be significantly higher in lichen planus (81 cases) as compared to the control group (80 subjects). There were no significant differences between the levels of DS in lichen planus patients and the control group. The authors suggested that increased levels of thiols could contribute to cell proliferation and lesion progression in lichen planus [7]. TDHPs were also investigated in fungal infections caused by *Malassezia* spp., a representative microorganism of the normal human skin flora, which, under certain circumstances, becomes a pathogen. In a study that included patients with tinea versicolor (42 cases) and a control group (36 cases), similar levels of NT and DS were found. According to this study, thiol metabolism seems not to be involved in the pathogenesis of tinea versicolor [25].

Based on the aforementioned results, it is important to note that thiols, their oxidation state, and regeneration are important for the normal cell functions and changes in these processes cause the development of various skin pathologies. Thiol deficiency induces increased susceptibility to oxidative stress, and the resulted damage is considered a key step in the onset and progression of several dermatological diseases [8,26,29]. In contrast, high levels of thiols were observed in the development of proliferative skin diseases [7,22].

## 5. Conclusions

The results of this study offer data on the role of TDH in the pathogenesis of chronic spontaneous urticaria. The lower levels of thiols determined before treatment in patients with chronic spontaneous urticaria and the higher levels measured after treatment could be a consequence of ROS generation due to chronic inflammation. Detecting high levels of DS in the pre-therapeutic phase in patients with urticaria shows that thiol oxidation is an early event in the pathogenesis of urticaria. The tendency of DS to normalize in patients with chronic urticaria during the treatment with H1-antihistamines was associated with symptom relief (reduction or disappearance of pruritus and urticarial plaques). These results suggest the involvement of TDH in the defense against the harmful effects of ROS in patients with chronic spontaneous urticaria. To the best of our knowledge, this is the first study investigating TDHPs in patients with chronic urticaria before and after treatment.

## Figures and Tables

**Table 1:** The characteristics of the patients with chronic spontaneous urticaria.

Patient Characteristic	Value
Age (years)—mean ± SD Female/male ratio	29.73 ± 3.19 1.5/1
History of angioedema	33.3%
Disease duration (months)—mean ± SD	9.2 ± 3.4

SD = standard deviation.

**Table 2:** Correlations between UAS and TDHPs in patients with chronic spontaneous urticaria, before and after treatment.

Parameter	Before Treatment *n* = 30		After Treatment *n* = 26	
	rho	*p*	rho	*p*
NT	−0.74	0.0001	−0.85	0.0001
TT	−0.73	0.0001	−0.80	0.0001
DS	0.10	0.58	0.01	0.9
DS/NT	0.46	0.009	0.41	0.02
DS/TT	0.46	0.008	0.43	0.02
NT/TT	−0.47	0.007	−0.43	0.02

NT—native thiol, TT—total thiol, DS—disulfides.

## Data Availability

The data that support the findings of this study are available on request from the corresponding author.
